# Methodology for three-dimensional analysis of asymmetries in joint moments in cycling

**DOI:** 10.3389/fbioe.2025.1692531

**Published:** 2026-01-07

**Authors:** Ezequiel Martín-Sosa, Juana Mayo, Joaquín Ojeda

**Affiliations:** 1 Departamento de Ingeniería Minera, Mecánica, Energética y de la Construcción, Escuela Técnica Superior de Ingeniería, Universidad de Huelva, Huelva, Spain; 2 Departamento de Ingeniería Mecánica y Fabricación, Escuela Técnica Superior de Ingeniería, Universidad de Sevilla, Seville, Spain

**Keywords:** 3D asymmetries analysis, 3D joint moment, cycling, normalized symmetry index, workload effect

## Abstract

**Background and introduction:**

The assessment of lower-limb joint moment asymmetries during cycling is critical, as inter-limb imbalances may lead to performance decrements, overload, or injury risk. While most investigations have focused on the sagittal plane, asymmetries may also arise in the frontal and transverse planes, with potential implications for both performance and health. The present study performed a three-dimensional analysis of joint moment asymmetries and examined the influence of pedalling power on their magnitude in ten amateur cyclists under three power conditions.

**Methodology:**

Asymmetries were quantified using a modified version of the Normalised Symmetry Index (NSI), the Cross-Correlation Coefficient (CCC), and the newly proposed metric Asymmetries During Cycle (ADC) index.

**Results:**

Results indicated that these indices must be applied jointly to identify whether asymmetries arise from magnitude differences, temporal pattern discrepancies, or both. The greatest asymmetries were observed in the frontal and transverse planes, and their magnitude decreased progressively with increasing pedalling power.

**Discussion:**

The novelty of this work resides in the combined application of NSI, CCC, and the ADC index to three-dimensional joint moment analysis, which together provide a comprehensive and mechanistic understanding of asymmetries throughout the pedalling cycle, an approach not previously reported in cycling biomechanics.

## 1 Introduction

Lower-limb asymmetries between dominant and non-dominant sides are common in daily activities. In sports such as cycling, these asymmetries may compromise performance and increase injury risk. Cycling involves highly repetitive movements, where even minor differences in strength, range of motion, or muscle activation may result in significant cumulative effects, leading to biomechanical inefficiencies, increased energy expenditure, and uneven joint and soft tissues loading. These factors can contribute to overuse injuries and chronic conditions in both amateur and professional athletes (Bini and Diefenthaeler, 2010; Smak et al., 1999).

A considerable number of studies related to the analysis of asymmetries in cycling practice have been reported in the scientific literature. Regarding the analysis of kinematic variables, previous studies have examined differences in hip, knee and ankle joint motion under varying workloads, as well as the evolution of 3D kinematic patterns in professional cyclists (Edeline et al., 2004; Pouliquen et al., 2018). Similarly, most research addressing kinetic variables has focused on pedal forces, crank torque and power distribution between legs under different testing conditions (Smak et al., 1999; Carpes et al., 2007; Carpes et al., 2007b; Bini and Hume, 2014; Bini et al., 2016; Bini et al., 2017; Farrel and Neira, 2023). These works collectively indicate that inter-limb asymmetries are commonly present across a range of cycling intensities and protocols. Although no studies have analysed joint-moment asymmetries between dominant and non-dominant legs, this gap is notable given the influence of these variables across sagittal, frontal and transverse planes on joint and muscle overload (Gardner et al., 2015; Fang et al., 2016; Shen et al., 2018). Since joint overload is difficult to estimate directly, assessing joint or muscle asymmetries in a 3D context can help approximate overload thresholds and thus prevent them. Quantifying the level of asymmetry is therefore essential for this methodology.

The Asymmetry Index (AI) is commonly employed when asymmetries in cycling are calculated (Cuk et al., 2001; Patterson et al., 2010). Despite its widespread use, it was demonstrated by Pouliquen et al. (2018) that this ratio is suitable only for evaluating asymmetries at specific moments within the pedalling cycle, namely, those in which the variables reach their peak values. If asymmetries are to be assessed at each instant of the pedalling cycle, this ratio presents certain limitations, particularly when the variables under study approach values near zero. For this reason, in one of the published studies, this author (Pouliquen et al., 2018) proposed the use of the Normalised Symmetry Index (NSI) defined by Gouwanda and Senanayake (2011) for analysing lower limb asymmetries during cycling activity.

Recent studies have emphasised the importance of evaluating asymmetries across the entire pedalling cycle using continuous and vector-based methods. Pouliquen et al. (2018) analysed three-dimensional joint-angle asymmetries under two pedalling power conditions, employing both the NSI and the Cross-Correlation Coefficient (CCC) to characterise differences in magnitude and timing. Their work demonstrated that asymmetries in the frontal and transverse planes were more sensitive to changes in workload and that the hip joint showed the highest levels of asymmetry. Da Silva Soares et al. (2021) examined asymmetries in the torque applied by each leg to the crank using both scalar variables (maximum torque) and vector-based functional data analysed through Functional ANOVA. Although no direct interaction between power and asymmetry was found, the study identified the specific instants within the cycle at which asymmetries emerged. Martín-Sosa et al. (2022) evaluated asymmetries in the three components of pedal force at each instant of the pedalling cycle using NSI and CCC. They also introduced the Asymmetries During Cycle (ADC) index, defined as the percentage of the cycle during which NSI exceeds a prescribed threshold. Their findings revealed marked asymmetries particularly in the mediolateral force component, previously associated with knee overload, and showed that combining NSI and CCC enables the distinction between asymmetries driven by differences in force magnitude, temporal patterning, or both.

Most studies evaluating asymmetries based on the AI and analysing the effect of power tend to employ ANOVA using scalar variables. Other investigations, such as the one conducted by da Silva et al., have assessed the effect of power on the pattern and magnitude of torque applied by each leg to the crank at each instant through Functional ANOVA (FANOVA). However, according to the current knowledge available to the authors, an assessment of how power affects the degree of asymmetry at every instant of the pedalling cycle has not yet been performed. One tool that enables this type of analysis is Statistical Parametric Mapping (SPM), which has already been utilised in the field of cycling by previous studies (Bini, 2021; Galindo-Martínez et al., 2021; Pouliquen et al., 2021; Park et al., 2022; Bing et al., 2024; Martín-Sosa et al., 2025). Bini (2021), examined how changes in saddle height affected knee joint kinematics, moments and forces in the sagittal and transverse planes. Galindo-Martínez et al. (2021) applied SPM to analyse the influence of fatigue on the three-dimensional kinematics of the lower limbs and trunk. Park et al. (2022) extended the method to joint angles, joint moments and muscle forces in the sagittal plane to assess the effect of crank length in standing cycling. More recently, Pouliquen et al. (2021) and Bing et al. (2024) used SPM to investigate the effects of fatigue and workload or saddle height on muscle forces. The most recent of these studies was conducted by Martín-Sosa et al. (2025), who examined the effect of pedalling power on three-dimensional joint moments at the hip, knee and ankle. In this work, SPM was applied to identify the specific intervals of the pedalling cycle in which significant differences between power levels occurred.

To address these gaps, this study aims to provide a comprehensive three-dimensional analysis of joint moment asymmetries during cycling and to evaluate the influence of pedalling power on their magnitude and temporal pattern. Asymmetries are assessed using a modified, the ADC index, both proposed by the authors, and the CCC. The effect of pedalling power is examined through SPM and ANOVA applied to scalar variables. This study introduces a methodological innovation: the integrated use of NSI, CCC, and ADC. NSI quantifies instantaneous magnitude differences, CCC evaluates temporal similarity, and ADC determines the proportion of the cycle affected by asymmetry. This combined framework enables the identification of whether asymmetries arise from magnitude differences, timing discrepancies, or both, providing a more complete biomechanical perspective.

Based on these considerations, the following hypotheses were formulated:

H1 Three-dimensional analysis is essential: Restricting asymmetry evaluation to the sagittal plane does not provide a complete picture; frontal and transverse planes must be included for performance and injury prevention.

H2 Temporal evolution matters: Assessing asymmetries across the entire pedalling cycle offers more detailed insights than focusing on isolated peak values.

H3 Origins of asymmetry are multifactorial: Differences in joint moments cannot always be explained by magnitude alone; pattern and timing discrepancies also contribute.

H4 Pedalling power influences asymmetry: Increasing pedalling power reduces the degree of asymmetry in joint moments.

To validate these hypotheses, the primary objective of this study is to perform a comprehensive analysis of asymmetries in three-dimensional joint moments and to investigate the biomechanical mechanisms underlying their occurrence. This approach represents an advancement over traditional methods that focus exclusively on the sagittal plane because of two reasons. First, it incorporates information from all three planes to provide a holistic understanding of joint behaviour. Second, it performs an analysis of the entire pedalling cycle, rather than focusing solely on its characteristic points. For this purpose, asymmetries will be assessed using a modification of the NSI proposed in this work, the CCC and the ADC index.

As a secondary objective, the effect of pedalling power on the occurrence of asymmetries in joint moments will also be analysed. To this end, statistical analyses will be conducted employing both SPM methodology and ANOVA with scalar variables.

## 2 Materials and methodology

### 2.1 Participants

To achieve the objectives established in this study, 10 participants were analysed, all men, adults, with a mean age of 26.87 ± 4.97 years, a mean height of 1.74 ± 0.07 m, a mean weight of 67.89 ± 11.34 kg and a mean BMI of 22.33 ± 3.18 kg/m^2^. The participants were volunteers who used the bicycle mainly for their daily commute to work, between 30 and 90 min per day, depending on their route, although they could use it recreationally on occasion. None of them undertook structured cycling training aimed at improving sporting performance. The sample size was based on previous studies found in the literature (Mornieux et al., 2007; Carpes et al., 2007b; Bini and Diefenthaeler, 2010; Bini et al., 2016; Pouliquen et al., 2018; Bini, 2021; Pouliquen et al., 2021; Martín-Sosa et al., 2025). Each participant was asked about their dominant leg, such as the leg used to kick a ball (Smak et al., 1999). The inclusion-exclusion criteria were as follows:

Inclusion criteria:

- Participants aged 18 years or older.
- Leg length discrepancy (dysmetria) less than or equal to 5 mm.
- Body size compatible with the test bicycle, assessed by measuring the inseam of each participant and confirming that it fell within the range recommended by the manufacturer for the frame size.
- Intrinsic Q-factor similar to the standard Q-factor of the bicycle.

Exclusion criteria:

- Diagnosed locomotor or cardiopulmonary conditions that could interfere with test performance.
- High-performance or professional cyclists.

To assess the discrepancy between lower limbs, each lower limb was measured using the methodology developed by Davis et al. (1991) which is used by other authors in the cycling biomechanics analysis (Bini et al., 2016). Participants signed a consent form. The study was approved by the Andalusian Biomedical Research Ethics Coordinating Committee (code number 0230-N-22).

### 2.2 Instrumentation

A three-dimensional motion capture system composed of twelve infrared cameras (Vicon Motion Systems Ltd., Oxford, United Kingdom) was employed. Half of the devices corresponded to the MXT010 model, with a maximum sampling capacity of 250 Hz, and were positioned at a height of approximately 3 m, at a distance of 2.5–3 m from the calibrated volume. The remaining cameras were of the Bonita model, featuring a compact format and a limit of 100 Hz, and were located at floor level, approximately 1.5 m from the cyclist. The acquisition frequency of the system was ultimately restricted to the lowest of the camera values, 100 Hz. All devices were synchronised using a Giganet unit, which also managed data transfer to the acquisition computer.

For the trials, a conventional road bicycle (Orbea H50) was used, fixed on a resistance roller (Elite Novo Force; Elite S.R.L., Italy). Clipless pedals were installed and cycling shoes with compatible cleats were provided to the participants. The position of the cleats was individually adjusted following the procedure described by Bini and Carpes (2014), with the aim of aligning the pedal axle with the region of highest plantar pressure. This alignment was assessed through pedalling at low cadence and high resistance prior to data capture. If deviations from the optimal position were detected, adjustments were made followed by a new verification. All equipment interventions were performed by the same investigator to ensure consistency.

### 2.3 Experimental data post-processing

Experimental data were collected and processed with Vicon Nexus® 2.12.1 commercial software. Based on residual analysis (Winter, 2009), the data were filtered with a low-pass filter (6 Hz, 4th order Butterworth filter) by a custom-made routine using Matlab R2024a (The MathWorks, Inc., Natick, MA, United States) to eliminate high-frequency noise (Fang et al., 2016; Gregersen and Hull, 2003).

**FIGURE 1:**
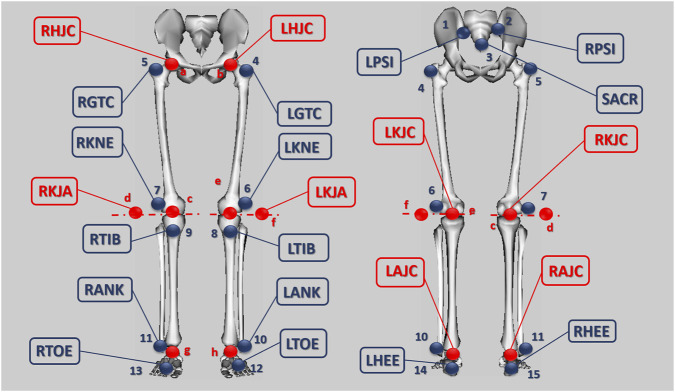
Location of markers that form the protocol. Blue: physical markers. Red: virtual markers.

Lower limb kinematics were obtained following the marker protocol proposed by Martín-Sosa et al. (2025), which considers a model of seven rigid segments (pelvis, thighs, shanks, and feet, bilaterally). Physical markers were placed on key anatomical prominences (e.g., posterior superior iliac spines, greater trochanter, lateral epicondyle, tibial tuberosity, lateral malleolus, calcaneus, and first metatarsal), complemented by virtual markers to estimate hip joint centres (Ehrig et al., 2006), ankle joint centres (Davis et al., 1991), and the instantaneous axis of rotation of the knee (Ehrig et al., 2007). In total, 15 physical markers and 8 virtual markers were used; a schematic of marker locations is shown in Figure 1. The model was developed without imposing joint constraints.

Pedal reaction forces were recorded using equipment installed on both pedals, previously developed by Martín-Sosa et al. (2021). These devices exhibited a measurement error below 4%, comparable to systems used in other similar studies (Balbinot et al., 2014).

Based on kinematic and force data, the inverse dynamics problem was solved using the Newton-Euler approach, following a bottom-up strategy. Joint moments were expressed in the local coordinate system of the proximal segment of each joint.

### 2.4 Test conditions

The pedalling conditions (power and cadence) were chosen according to the participants physical condition to avoid the fatigue appearance shortly after the test started. The cadence was set at approximately 90 rpm and controlled by acoustic signals. To analyse the effect of power on asymmetries, three absolute pedalling power levels were selected: 170 W (P1), 240 W (P2) and 310 W (P3), which were adjusted by modifying the friction of the roller on the rear wheel. These three power levels (170 W, 240 W, and 310 W) were selected to represent low, intermediate, and high pedalling efforts typical of recreational cycling. The selection was informed by preliminary tests with a subset of participants, who tested a range of powers and chose levels that matched three specific conditions: a power similar to their typical daily commuting effort (240 W, P2), a comfortable low-intensity power (170 W, P1), and a demanding but sustainable high-intensity power (310 W, P3) that would not induce extreme fatigue after 10–15 min of pedalling. This approach ensured that the study assessed asymmetries under realistic cycling conditions without incorporating protocols designed to induce fatigue.

As part of the assessment, resting heart rate and peripheral oxygen saturation were measured using a pulse oximeter, and cardiopulmonary auscultation was carried out. Participants also completed a basic health questionnaire, which included questions on previous injuries and relevant medical history. The maximum heart rate of each participant was determined separately. To evaluate exercise-induced breathlessness, the Medical Research Council (MRC) dyspnoea scale was used, as it is particularly well suited for use in non-athletic populations.

In the present study, saddle height was determined following the methodology proposed by Holmes et al. (1994), which is based on the measurement of knee flexion in static conditions. However, given that dynamic assessments were conducted, this method was adapted in accordance with the recommendations of Ferrer-Roca et al. (2012) and Ferrer-Roca et al. (2014), allowing for accurate application under dynamic conditions.

To optimise individual comfort, minor adjustments to saddle height were permitted, ensuring in most cases that the minimum knee flexion angle remained within the established reference range (Holmes et al., 1994; Ferrer-Roca et al., 2012; Ferrer-Roca et al., 2014). Participants whose minimum knee flexion angles, after these adjustments, deviated from the accepted range were excluded from the study. Such deviations were indicative of an inappropriate bicycle frame size, and this criterion was explicitly considered within the inclusion and exclusion parameters of the study.

A single saddle model was employed across all participants throughout the data collection process. This saddle, originally provided with the bicycle, had a width of 16 cm at its widest point and 5 cm at its narrowest, and featured an anatomical cut-out designed to alleviate perineal pressure. The decision to standardise the saddle was informed by prior studies on bicycle fitting (Kotler et al., 2016; Cyr and Ascher, 2023), which underscore the importance of saddle geometry, particularly in prolonged cycling sessions. However, considering that the duration of cycling in the present study was limited to 10–15 min, the risk of discomfort attributable to saddle design was minimised. Nevertheless, subjective comfort of participants was continuously monitored through verbal feedback obtained before, during, and after testing, with no reports of saddle-related discomfort being recorded.

Each test session consisted of two distinct phases. The initial phase, lasting 2–3 min, served as both a warm-up period and an opportunity to confirm participant comfort with the assigned saddle height and bicycle configuration. Following this acclimatisation period, the second phase commenced, during which the motion capture of pedalling was performed. To minimise the influence of muscular fatigue on biomechanical data, five recordings of 10 s each were obtained per participant, with rest intervals of approximately 5–10 s between trials.

### 2.5 Analysis of asymmetries

Asymmetries analysis between the dominant and non-dominant leg for each variable was carried out by applying three metrics to the data collected: firstly, the NSI (Gouwanda and Senanayake, 2011), which is a modification of the Symmetry Ratio implemented by Patterson et al. (2010). Secondly, the CCC developed by Li and Caldwell (1999). Finally, the ADC index introduced by our research team (Martin-Sosa et al., 2022).

#### 2.5.1 Normalised symmetry index

The NSI was used to determine the degree of asymmetry at each instant of the pedal cycle between the dominant and non-dominant side (Gouwanda and Senanayake, 2011).

$$NSI\left(t\right)=\frac{{JMN}_{D}\left(t\right)-{JMN}_{ND}\left(t\right)}{0.5*\left({JMN}_{D}\left(t\right)+{JMN}_{ND}\left(t\right)\right)}*100\%$$ (1)

$${JMN}_{i}\left(t\right)=\frac{{JM}_{i}\left(t\right)-{JM}_{\min}}{{JM}_{\max}-{JM}_{\min}}+1$$ (2)

In Equation 1, *NSI(t)* represents the normalised rate of symmetry between dominant and non-dominant leg for each time instant *t*. ${JMN}_{D}\left(t\right)$ and ${JMN}_{ND}\left(t\right)$ are the normalised values for the joint moments corresponding to instant *t* for the dominant and non-dominant leg respectively. Normalisation prevents possible singularities from appearing when force values tend to zero. The normalised variables are defined in Equation 2, where ${JM}_{i}\left(t\right)$ is the value of the raw variable for time instant *t* and leg *i*, where *i* is either leg dominant (D) or non-dominant (ND). In the study proposed by Gouwanda and Senanayake (2011), the minimum and maximum values defined in Equation 2 were referenced to the right leg in all cases. This normalisation of variables is valid when the level of asymmetry is low, in which case the value of the normalised variables remains within the interval [1, 2]. However, if the maximum value for the left leg exceeds that of the right leg, the normalised variable may exceed a value of 2. Conversely, if the minimum value for the left leg is lower than that of the right leg, the normalised variable may fall below 1 and may even become negative. In both scenarios, the intended purpose of variable normalisation is compromised. Furthermore, if the normalised variable becomes negative, singularities may arise in the estimation of the NSI (Equation 1).

##### 2.5.1.1 Modification to the NSI proposed in this work

For all the reasons outlined above, this study proposes a modification to the definition of the normalised variables. The modification in the calculation of the NSI performed in this work, compared to that of Gouwanda and Senanayake (2011), consists in defining a new general normalized variable ${JMGN}_{i}\left(t\right)$ (see Equation 3) where ${JM}_{gmax}$ as the maximum absolute value obtained between the dominant and non-dominant leg during the same analysed cycle. Similarly, ${JM}_{gmin}$ corresponds to the minimum absolute value recorded in that cycle, regardless of whether it originates from the dominant or non-dominant leg.

$${JMGN}_{i}\left(t\right)=\frac{{JM}_{i}\left(t\right)-{JM}_{gmin}}{{JM}_{gmax}-{JM}_{gmin}}+1$$ (3)

In this work, a NSI threshold value of NSI 10% has been adopted, above which a state of asymmetry between dominant and non-dominant leg components is considered (Carpes et al., 2007b).

#### 2.5.2 Asymmetries during cycle

The *ADC* index (Martin-Sosa et al., 2022) has been calculated, which is defined in Equation 4:

$$ADC=\frac{t_{\left|NSI\right|>10\%}}{T}*100$$ (4)

Where T is the duration of a pedalling cycle and $t_{\left|NSI\right|>10\%}$ the time where the index NSI is higher than 10% in modulus. This index can be used to determine the percentage of the pedal cycle in which a component of the force behaves asymmetrically. Alongside the *ADC*, the *NSI*_*mean*_ value for each participant was also calculated, representing the average value of each NSI curve obtained.

#### 2.5.3 Cross-correlation coefficient

The CCC (Li and Caldwell, 1999) was used to detect similarities in the temporal evolution of the variables obtained for the dominant and non-dominant legs. It is defined as follows:

$$CCC\left(k\right)=\frac{c_{{JM}_{D}{JM}_{ND}}\left(k\right)}{\sqrt{\sum_{t=1}^{N}{\left({JM}_{D}\left(t\right)-\bar{{JM}_{D}}\right)}^{2}*\sum_{t=1}^{N}{\left({JM}_{ND}\left(t\right)-\bar{{JM}_{ND}}\right)}^{2}}}$$ (5)

Where:

$$c_{{JM}_{D}{JM}_{ND}}\left(k\right)=\left\{\begin{array}{c} A_{{JM}_{D}{JM}_{ND}}\left(k\right)+B_{{JM}_{D}{JM}_{ND}}\left(k\right),if\;k\in \left[1,N\right]\\ A_{{JM}_{D}{JM}_{ND}}\left(k\right),if\;k=0 \end{array}\right.$$ (6)

Where:

$$A_{{JM}_{D}{JM}_{ND}}\left(k\right)=\sum_{t=1}^{N-k}\left({JM}_{D}\left(t\right)-\bar{{JM}_{D}}\right)*\left({JM}_{ND}\left(t+k\right)-\bar{{JM}_{ND}}\right)$$ (7)

$$B_{{JM}_{D}{JM}_{ND}}\left(k\right)=\sum_{t=N-k+1}^{N}\left({JM}_{D}\;\left(t\right)-\bar{{JM}_{D}\;}\right)*\left({JM}_{ND}\;\left(t-N+k\right)-\bar{{JM}_{ND}\;}\right)$$ (8)

In Equation 5, *CCC(k)* represents the cross-correlation coefficient for a time shift *k* between the dominant and non-dominant side signal. For this purpose, the measurements of the signals applied on the non-dominant side were put in phase with the signals applied on the dominant side to synchronise both signals at the start of the pedal cycle, top dead centre (TDC). For the set of equations (Equations 5‐8), ${JM}_{D}\left(t\right)$ and ${JM}_{ND}\left(t\right)$ represent the joint moments obtained from the dominant and non-dominant leg at each instant of time *t*. *N* is the number of instants that compose the pedalling cycle. $\bar{{JM}_{D}}$ and $\bar{{JM}_{ND}}$ represent the average value of the joint moments obtained by the dominant and non-dominant leg over the analysed pedalling cycle, respectively. $\tau_{lag}$ is defined as the pedal angle delay and indicates the lag of the non-dominant component with respect to the dominant component. It corresponds to the value of *k* at which the correlation coefficient *CCC (k)* is maximum, *CCC*_*max*_. A $\tau_{lag}$ positive value indicates that the non-dominant leg component is shifted forward in the pedal cycle relative to the dominant leg. The *CCC (k)* oscillates in the interval [−1, 1], with *CCC*_*max*_ corresponding to the best overlap between the joint moment on the dominant side and the non-dominant side. The *CCC (k = 0)* will give an indication of pattern similarity between the two sets of data. If there was a time (or phase) shift between two-time series that have similar patterns, the magnitude of this shift could be found by assessing *CCC (k)* at different values of *k*. An objective measure of the time shift is the *k* at which *CCC (k)* is *CCC*_*max*_.

### 2.6 Data analysis

Once the results were obtained outliers were eliminated using the box-and-whisker method. The analysis of the results was performed by defining one factor, pedalling power, with three levels (P1, P2 and P3), both for the statistical analysis using SPM methodology and scalar variables. For each level, the variables analysed for the vectorial study were the temporal evolution of NSI in the three anatomical planes of the three joints forming the lower limb. For the scalar study, the variables analysed were the range, the maximum and the minimum value of the same NSI. In the scalar analysis, the effect of power on the *CCC*_*max*_, $\tau_{lag}$ , and *ADC* was also examined.

To study the effect of power on the vectorial variables analysed, a statistical parametric mapping analysis was carried out using the open source spm1d statistical package (www.spm1d.org), in Matlab® R2023a (The MathWorks, Inc., Natick, MA, United States). The SPSS® software was used to study the effect of the power on the scalar variables.

A similar approach was used for both types of variables. In a first step, the Shapiro-Wilk test was applied, and the normality hypothesis was fulfilled for all cases. Subsequently, homogeneity was analysed, which was only fulfilled for some variables. To determine whether pedalling power influenced the variables defined in this study, a comparison of means was initially carried out using a one-factor ANOVA analysis. A one-way repeated-measures analysis of variance (ANOVA) was employed to examine differences across experimental conditions within the same participants. This design inherently accounts for interindividual variability by treating each participant as their own control, thereby reducing between-subject error and enhancing statistical power. The model incorporates a term for within-subject variability, estimated from deviations of individual observations relative to each participant’s overall mean. Consequently, the residual error reflects only the unexplained variation after accounting for the experimental factor. Assumptions of sphericity were assessed using Mauchly’s test; when violated, degrees of freedom were adjusted using Greenhouse–Geisser or Huynh–Feldt corrections to maintain the validity of the F-statistic. Components that did not satisfy the homogeneity hypothesis were subjected to Welch’s test. In all studies the significance level was set at a value α = 0.05. Post hoc pairwise comparisons were conducted across all levels of the factor. To control for the familywise Type I error rate, p-values were adjusted using the Bonferroni correction. The reported p-values correspond to the Bonferroni-adjusted values, and differences were considered statistically significant at the adjusted α level. All analyses were performed using pairwise comparisons with Bonferroni adjustment. For cases of non-homogeneity, the Games-Howell *post hoc* test was applied. Finally, the effect size would be calculated using Cohen’s d (Cohen, 1988) defining a large effect size for a d value >0.8 (Bini, 2021).

## 3 Results

Table 1 presents the overall results from the asymmetry analysis at 240 W (P2). This intermediate power was selected as it represents a moderate effort, close to the typical commuting intensity of the participants, and allows the analysis to reflect general movement patterns without bias towards extreme efforts. The table includes the *CCC*_*max*_, which, as previously described, represents the value that maximises the similarity of the patterns between the curves obtained from the dominant and non-dominant legs, along with the $\tau_{lag}$ . Additionally, the table provides the average value of *NSI*_*mean*_ across all participants, as well as the mean *ADC*.

**TABLE 1:** Mean values and standard deviation (mean ± SD) across the 10 participants for *CCC*_*max*_, $\tau_{lag}$ , *NSI*_*mean*_, and *ADC* for the three joints analysed across the three anatomical planes.

Joint	Moment	*CCC*_*max*_	$\tau_{lag}$ (°)	*NSI*_*mean*_ (%)	*ADC* (%)
HIP	FL	0.829 ± 0.092	−21.983 ± 35.687	−8.162 ± 9.907	68.409 ± 13.187
	AD	0.808 ± 0.077	−2.813 ± 38.823	4.137 ± 4.441	64.251 ± 9.822
	INT	0.794 ± 0.088	−10.845 ± 30.072	−3.929 ± 7.585	71.577 ± 17.307
KNEE	FL	0.961 ± 0.022	−7.402 ± 8.068	7.598 ± 10.583	46.518 ± 16.493
	AD	0.861 ± 0.058	−14.648 ± 14.842	−0.665 ± 10.023	49.136 ± 11.861
	INT	0.793 ± 0.086	22.310 ± 52.612	2.617 ± 17.094	76.641 ± 9.327
ANKLE	DFL	0.948 ± 0.028	−8.874 ± 10.586	10.185 ± 15.089	43.985 ± 12.157
	INV	0.908 ± 0.032	−17.569 ± 11.470	2.161 ± 13.217	71.564 ± 20.347
	INT	0.937 ± 0.028	−5.370 ± 9.323	−5.849 ± 6.850	52.530 ± 20.640

Abbreviation: AD, Adduction; DFL, Dorsiflexion; FL, Flexion; INT, Internal rotation; INV, Ankle inversion.

As shown in Table 1, the ankle joint exhibits the highest *CCC*_*max*_ values across all three anatomical planes, with all *CCC*_*max*_ values exceeding 0.9. This joint also displays the lowest $\tau_{lag}$ values, all above −20°. In contrast, the hip joint consistently shows the lowest *CCC*_*max*_ values among the three joints analysed. Notably, internal rotation moments of both the hip and knee joints exhibit the lowest *CCC*_*max*_, both close to 0.79. Regarding the $\tau_{lag}$ values, both the hip and knee joints show substantial phase shifts between the dominant and non-dominant legs, with the hip flexion moment showing the highest $\tau_{lag}$ , nearly −22°.

With respect to the *ADC*, values above 40% are observed across all joints and anatomical planes. Once again, the hip joint stands out as the one exhibiting the highest *ADC* values overall, whereas the knee and ankle present relatively similar *ADC* values. The lowest *ADC* is observed in the ankle dorsiflexion moment (43.98%), while the highest value corresponds to the knee internal rotation moment (76.64%).

### 3.1 Asymmetries analysis methodology

**FIGURE 2:**
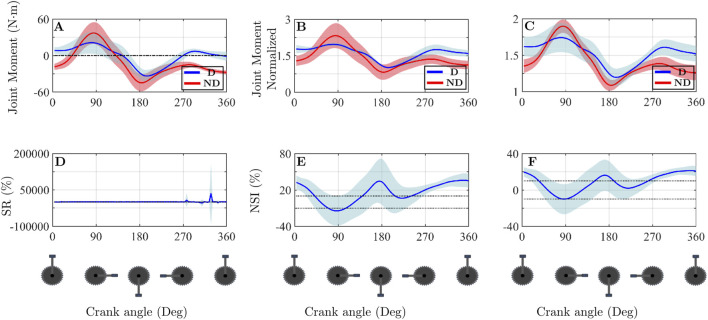
Differences in the calculation of normalised variables and in the asymmetry analysis of the hip flexion moment. **(A)** Joint moments for both limbs for one participant. **(B)** Normalised joint moments according to the methodology proposed by of Gouwanda and Senanayake. **(C)** Normalised joint moments following the methodology described in the present study. **(D)** Symmetry Ratio calculated as proposed by Patterson et al. **(E)** NSI calculated using the normalisation method of Gouwanda and Senanayake. **(F)** NSI calculated based on the methodology developed in this study. The thin dotted black lines appearing in figures **(D–F)** refer to the asymmetry threshold chosen in this article, which is ±10%. Abbreviations: D, Dominant side; ND, Non-dominant side.

**FIGURE 3:**
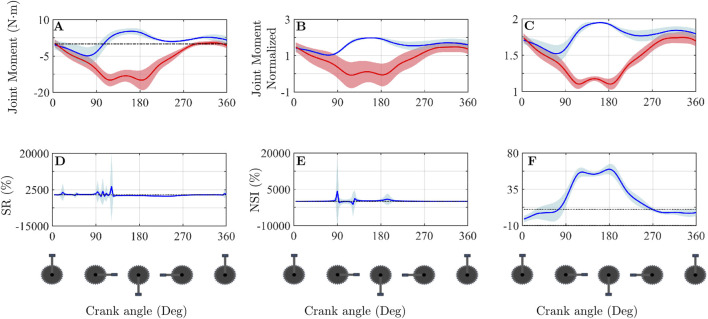
Differences in the calculation of normalised variables and in the asymmetry analysis of the hip internal rotation moment. **(A)** Joint moments for both limbs for one participant. **(B)** Normalised joint moments according to the methodology proposed by Gouwanda and Senanayake. **(C)** Normalised joint moments following the methodology described in the present study. **(D)** Symmetry Ratio calculated as proposed by Patterson et al. **(E)** NSI calculated using the normalisation method of Gouwanda and Senanayake. **(F)** NSI calculated based on the methodology developed in this study. The thin dotted black lines appearing in figures **(D–F)** refer to the asymmetry threshold chosen in this article, which is ±10%.

Figures 2, 3 show results of hip flexion and internal rotation moments, respectively. In both cases, Figures 2A, 3A show the raw hip moment values for the dominant (blue) and non-dominant (red) legs in one of the participants. Figures 2B, 3B present the normalised moments using the method proposed by Gouwanda and Senanayake (2011). Figures 2C, 3C show the normalisation applied in this study, as defined in Equations 1 and 2. Figures 2D–F, 3D–F illustrate different approaches for analysing asymmetries throughout the pedalling cycle. Figures 2D, 3D apply the Symmetry Ratio (SR) method developed by Patterson et al. (2010). Figures 2E,F display the NSI values calculated using the normalised data from Figures 2B,C, respectively. The same explanation applies to Figures 3E,F with respect to Figures 3B,C.

### 3.2 Asymmetries analysis

**FIGURE 4:**
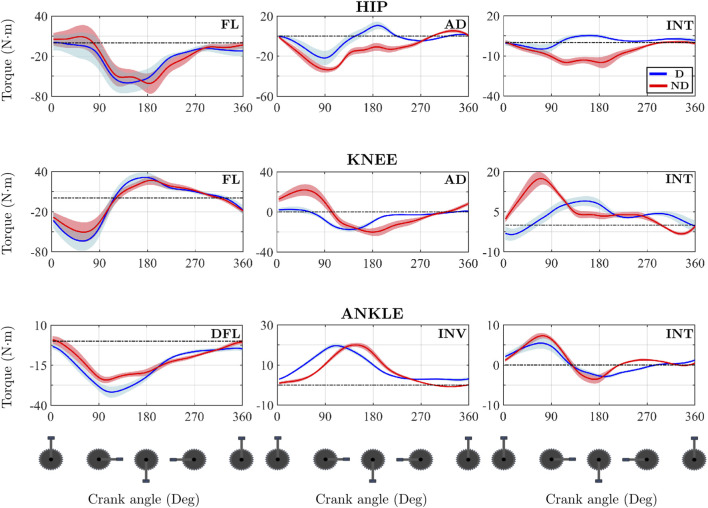
Mean temporal evolution of joint moments for both limbs for the three joints analysed across the anatomical planes for one representative participant (participant 3). Abbreviations: FL, Flexion; AD, Adduction; INT, Internal rotation; DFL, Dorsiflexion; INV, Ankle inversion.

**FIGURE 5:**
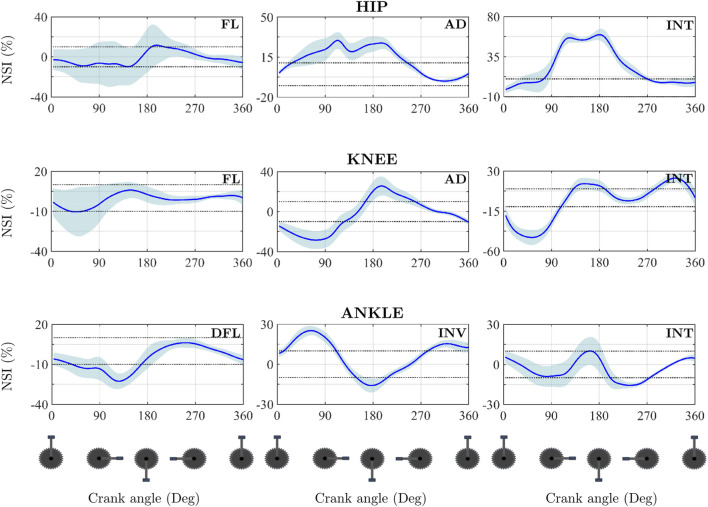
Mean temporal evolution of NSI for the three joints analysed across the anatomical planes for one representative participant. The thin dotted black lines refer to the asymmetry threshold chosen in this article, which is ±10%.

As the overall results showed high variability, participant 3 was used as the representative participant for the asymmetries analysis. This participant was selected for several reasons. Firstly, in the in-depth analysis this individual clearly exhibited all the asymmetry patterns identified in the sample. In addition, a complementary representativeness assessment based on the Z-scores of 81 scalar variables was conducted, showing that this participant fell within ±1 standard deviation of the group mean in approximately 80% of the parameters analysed. These results indicate that the participant displayed central characteristics within the cohort and can therefore be considered representative of the general profile of the sample. Figure 4 displays the time evolution of the three-dimensional joint moments for both legs in Participant 3. Figure 5 shows the patterns obtained by calculating the NSI for the same participant. Table 2 includes the *CCC*_*max*_, $\tau_{lag}$ and *ADC* values for each comparison. It should be noted that the analysis of this participant is included solely for illustrative purposes and does not replace or condition the group-level findings, which form the basis of the conclusions.

**TABLE 2:** Mean values and standard deviation of *CCC*_*max*_, $\tau_{lag}$ and *ADC* for the three joints analysed in the three anatomical planes for participant 3.

Joint	Moment	*CCC*_*max*_ ± SD	$\tau_{lag}$ (°) ± SD	*ADC* (%)
HIP	FL	0.896 ± 0.059	−9.299 ± 19.984	51.00 ± 13.29
	AD	0.658 ± 0.133	−5.699 ± 7.895	54.17 ± 7.88
	INT	0.691 ± 0.143	−82.501 ± 83.380	58.50 ± 10.14
KNEE	FL	0.972 ± 0.019	−0.900 ± 6.887	22.25 ± 15.87
	AD	0.848 ± 0.064	−34.200 ± 6.235	59.83 ± 7.23
	INT	0.883 ± 0.032	66.000 ± 9.626	61.67 ± 9.02
ANKLE	DFL	0.948 ± 0.017	−11.099 ± 7.434	37.08 ± 13.63
	INV	0.991 ± 0.004	−30.000 ± 4.158	62.33 ± 6.15
	INT	0.909 ± 0.026	−0.900 ± 7.538	38.50 ± 8.16

In the sagittal plane, the knee flexion moment exhibited a high degree of bilateral similarity (*CCC*_*max*_ = 0.972) with a negligible phase shift $\tau_{lag}$ = −0.9°), resulting in an NSI that remained largely within the ±10% symmetry threshold throughout the cycle. Conversely, the hip internal rotation moment presented a low correlation (*CCC*_*max*_ < 0.7) and a substantial phase shift ( $\tau_{lag}$ = −82.5°). Consequently, the NSI values consistently exceeded the 10% threshold, indicating that the dominant leg produced higher moment values throughout the majority of the cycle.

Regarding the ankle joint, the inversion moment demonstrated the highest pattern similarity (*CCC*_*max*_ = 0.991) and comparable magnitudes between limbs (Figure 4). However, the NSI frequently exceeded ±20% (Figure 5). This discrepancy was associated with a notable temporal lag $\tau_{lag}$ = −30°), indicating that the dominant leg’s moment preceded that of the non-dominant leg. In contrast, the ankle internal rotation moment showed high pattern similarity (*CCC*_*max*_ = 0.909) and minimal lag $\tau_{lag}$ close to 0°), yet the NSI still surpassed the threshold at specific cycle phases due to distinct magnitude differences (Figure 4). Finally, the ankle dorsiflexion moment reflected a combination of these factors: despite a high *CCC*_*max*_ (0.948), a phase shift of −11° combined with differences in peak magnitudes resulted in NSI values falling below −10% for approximately 35% of the cycle (from 15° to 50°).

### 3.3 Workload effect in asymmetries

**FIGURE 6:**
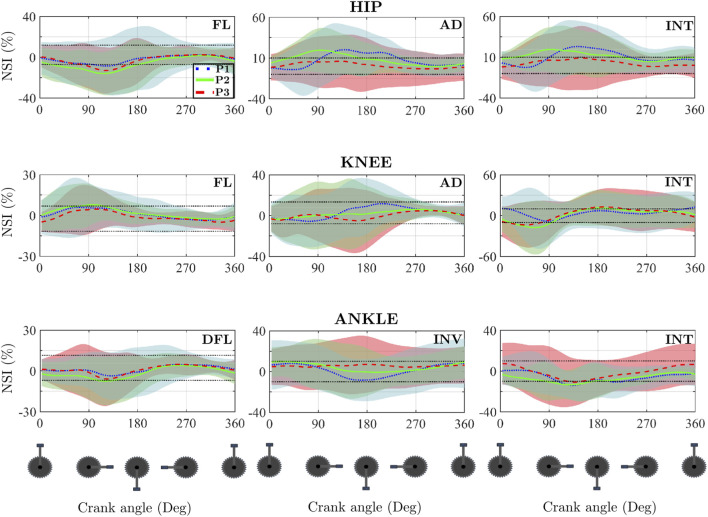
Temporal evolution of average NSI for the 10 participants at the different powers studied, in the three joints studied and in the three anatomical planes. The thin dotted black lines refer to the asymmetry threshold chosen in this article, which is ±10%.

Figure 6 presents the average temporal evolutions of the NSI for the 10 participants analysed across the three pedalling power outputs. This figure displays the NSI values for the three joints comprising the lower limb and for each of the three anatomical planes. These curves were used to examine the effect of pedalling power on the NSI using the SPM methodology. However, no statistically significant differences were found.

Table 3 summarizes the statistical analysis of the effect of pedalling power on asymmetry indices. The SPM analysis of the vectorial NSI evolution did not yield statistically significant differences across power conditions (Figure 6). However, the analysis of scalar variables revealed significant effects in specific parameters.

**TABLE 3:** Mean and standard deviation of the *CCC*_*max*_, $\tau_{lag}$ (°) and *ADC* (%) value for the 3 powers output and *p-value* for the different statistical analysis.

Variable			P1	P2	P3	*P*
HIP	FL	*CCC*_*max*_	0.780 ± 0.080	0.829 ± 0.093	0.879 ± 0.101	0.070
		$\tau_{lag}$	−8.46 ± 34.68	−22.01 ± 20.99	−14.80 ± 31.08	0.596
		*ADC*	68.41 ± 13.19	55.57 ± 16.77	48.90 ± 16.61	**0.029** ^*^
	AD	*CCC*_*max*_	0.751 ± 0.088	0.804 ± 0.145	0.810 ± 0.180	0.598
		$\tau_{lag}$	−42.79 ± 48.10	−4.79 ± 37.81	5.66 ± 34.45	**0.031** ^*^
		*ADC*	64.25 ± 9.82	53.93 ± 14.60	60.29 ± 21.77	0.368
	INT	*CCC*_*max*_	0.766 ± 0.091	0.794 ± 0.148	0.844 ± 0.131	0.384
		$\tau_{lag}$	−29.68 ± 44.62	−8.74 ± 42.96	5.55 ± 70.78	0.359
		*ADC*	71.58 ± 17.31	60.16 ± 17.70	58.61 ± 17.86	0.218
KNEE	FL	*CCC*_*max*_	0.918 ± 0.038	0.961 ± 0.018	0.970 ± 0.018	**<0.001** ^#*^
		$\tau_{lag}$	−9.14 ± 16.12	−7.45 ± 9.65	−9.34 ± 16.66	0.950
		*ADC*	46.52 ± 16.49	30.25 ± 11.54	28.61 ± 16.17	**0.022** ^*^
	AD	*CCC*_*max*_	0.857 ± 0.106	0.857 ± 0.112	0.877 ± 0.090	0.880
		$\tau_{lag}$	−16.51 ± 60.76	−12.26 ± 73.24	4.67 ± 41.54	0.708
		*ADC*	49.14 ± 11.86	53.93 ± 13.05	45.28 ± 11.22	0.291
	INT	*CCC*_*max*_	0.740 ± 0.150	0.790 ± 0.062	0.825 ± 0.086	0.218
		$\tau_{lag}$	6.99 ± 61.38	22.98 ± 8.45	21.37 ± 71.52	0.848
		*ADC*	76.54 ± 9.33	75.66 ± 13.22	73.05 ± 16.65	0.827
ANKLE	DFL	*CCC*_*max*_	0.942 ± 0.023	0.949 ± 0.036	0.958 ± 0.033	0.510
		$\tau_{lag}$	−5.48 ± 9.53	−8.84 ± 8.36	−8.72 ± 32.83	0.916
		*ADC*	43.98 ± 12.16	39.64 ± 13.03	35.20 ± 20.84	0.473
	INV	*CCC*_*max*_	0.898 ± 0.085	0.909 ± 0.147	0.975 ± 0.021	**0.034** ^*^
		$\tau_{lag}$	−9.95 ± 16.93	−16.60 ± 30.71	3.99 ± 17.45	0.135
		*ADC*	71.56 ± 20.35	65.99 ± 25.73	72.80 ± 20.73	0.771
	INT	*CCC*_*max*_	0.918 ± 0.038	0.932 ± 0.039	0.917 ± 0.050	0.699
		$\tau_{lag}$	−4.15 ± 26.12	−6.01 ± 22.05	−31.24 ± 53.22	0.198
		*ADC*	52.53 ± 20.64	43.04 ± 14.79	47.73 ± 24.14	0.583

^#^Significant differences between P1 and P2. *Significant changes from P1 to P3. ^†^Significant changes between P2 and P3. Bold values indicate statistical significance.

Significant reductions in asymmetry were observed with increasing power output in the sagittal plane. Specifically, the *ADC* for the hip flexion moment decreased significantly from 68.41% ± 13.19 at P1 (170 W) to 48.90% ± 16.61 at P3 (310 W) (p = 0.029). Similarly, the *ADC* for the knee flexion moment decreased from 46.52% ± 16.49 at P1 to 28.61% ± 16.17 at P3 (p = 0.022). Furthermore, the *CCC*_*max*_ for the knee flexion moment significantly increased with workload (p < 0.001), indicating greater bilateral pattern similarity at higher intensities.

In the non-sagittal planes, the *CCC*_*max*_ for the ankle inversion moment showed a significant increase between P1 and P3 (p = 0.034), approaching a value of 1. Additionally, the $\tau_{lag}$ for the hip adduction moment was significantly affected (p = 0.031), shifting from a negative value at P1 (−42.79° ± 48.10) to a positive value at P3 (5.66° ± 34.45), indicating a reversal in the temporal lead between the dominant and non-dominant legs.

## 4 Discussion

### 4.1 Methodological contribution

The precise quantification of asymmetries in cyclic movements requires robust metrics capable of managing the complex signal fluctuations inherent to 3D analyses. It was observed that both the standard AI and the NSI normalization proposed by Gouwanda and Senanayake (2011) are vulnerable to mathematical singularities when joint moments approach zero or when sign reversals occur between limbs, situations frequently present in the frontal and transverse planes of cycling. The normalization adjustment introduced in this study (Equation 3) effectively addressed these limitations, providing a consistent range [1, 2] and allowing stable NSI computation throughout the pedal cycle. This refinement is essential for future biomechanical investigations of continuous asymmetry in closed-loop movements, ensuring that peaks in asymmetry indices represent real biomechanical imbalances rather than mathematical artefacts.

### 4.2 Asymmetries analysis

This study focused on the analysis of functional asymmetries, defined as significant differences in strength, mobility, neuromuscular activation, or postural control between homologous body segments. Structural asymmetries, which arise from anatomical variations or morphological alterations, were excluded in accordance with the predefined inclusion and exclusion criteria. This distinction ensures that the observed differences are attributable to functional factors rather than underlying structural anomalies, thereby improving the interpretability and validity of the findings.

From the analysis of inter-limb asymmetries across hip, knee and ankle joint moments in all three anatomical planes, several relevant observations can be drawn. The high *CCC*_*max*_ values indicate that the moment patterns of both legs are generally very similar; however, the presence of temporal offsets reflected by $\tau_{lag}$ shows that asymmetries do not arise solely from differences in magnitude, but also from desynchronisation within the pedal cycle and variations in pattern shape. The interaction between pattern morphology, timing and magnitude differs across the joint moments examined, demonstrating that asymmetry does not have a single biomechanical origin.

The *ADC* index, which identifies the proportion of the cycle during which asymmetry exceeds a clinically relevant threshold, confirms that these differences occur throughout a substantial portion of pedalling, not only in the sagittal plane but also in the frontal and transverse planes. Interpreting *NSI*_*mean*_ can be misleading, as oscillations of the NSI around positive and negative values attenuate the true magnitude of asymmetry when averaged. Moreover, the lack of a universal reference pattern and the considerable intra- and inter-subject variability complicate global interpretation of the metrics. For this reason, the combined use of *CCC*_*max*_, $\tau_{lag}$ and *ADC* is essential for accurately identifying the different mechanisms underlying asymmetry.

Taken together, these metrics show that asymmetries may arise from differences in pattern morphology, temporal misalignment between limbs or discrepancies in moment magnitude, each with distinct implications for load distribution across the joints. Such differences may contribute to biomechanical imbalances and overload, consistent with previous reports on the influence of inter-limb asynchrony and variability on injury risk (Gardner et al., 2015; Fang et al., 2016; Shen et al., 2018). It is particularly noteworthy that the hip tends to exhibit the greatest asymmetries, a phenomenon also observed in earlier cycling studies (Pouliquen et al., 2018) and in other sports such as rowing (Buckeridge et al., 2015). This behaviour can be explained by the closed-chain nature of pedalling: asymmetries originating at the ankle or knee are often compensated proximally at the hip, although such compensations are not always biomechanically efficient and may increase the likelihood of overload (Pouliquen et al., 2018; Buckeridge et al., 2015).

Moreover, an appropriate definition of the closed kinematic chain formed by the cyclist and the bicycle, ensuring symmetry between the kinematic chains of the right and left legs, may help prevent potential functional asymmetries that could lead to overuse injuries (Belmonte et al., 2025). This can be achieved through proper adjustment of the bicycle’s parameters and correct posture on the bike. Other possible causes of functional asymmetries include poor pedalling technique or limited neuromuscular control (Visentini and Clarsen, 2016).

The emergence of such asymmetries may produce several effects. Firstly, asymmetric kinematic chains or inadequate pedalling technique may result in one leg becoming more dominant during the power phase, which can lead to overload of the patellar tendon and patellofemoral pain (Visentini and Clarsen, 2016). Secondly, asymmetries in hip internal rotation caused by pedalling technique or saddle position may alter pelvic kinetics, favouring iliotibial band syndrome and lumbar pain (Ménard et al., 2020). Finally, alterations in the activation patterns of knee extensors and abductors may cause asymmetries in force distribution across the joint, thereby contributing to patellofemoral pain and the persistence of symptoms (Dieter et al., 2014). In this context, the case of participant 3 is illustrative for understanding how these mechanisms can coexist within the same individual. The combination of similar but phase-shifted patterns in some joint moments, together with magnitude differences or pattern incongruences in others, suggests that asymmetries do not originate from a single source but rather from the interaction of multiple misalignment factors. This individual behaviour highlights that even cyclists with seemingly stable movement patterns may present complex imbalances, where simultaneous differences in pattern, phase and magnitude contribute to sustained asymmetry throughout the pedal cycle. This example reinforces the need for individualised analysis to identify the dominant cause of asymmetry and to understand its potential biomechanical implications.

### 4.3 Workload effect in asymmetries

The effect of workload on asymmetries appears to vary depending on the analytical approach used. When examining the temporal evolution of NSI via SPM, no statistically significant effects were observed, which is likely due to the high variability inherent to NSI patterns. This variability stems from the absence of a standardised reference pattern: each participant, regardless of cycling experience, presents a unique asymmetry profile influenced by their individual pedalling technique, producing considerable inter-subject variance.

The differences between ANOVA and SPM results can be explained by the distinct aspects of asymmetry captured by each method. ANOVA assesses overall or integrated magnitudes, as reflected in scalar metrics such as *ADC* and *CCC*_*max*_, while SPM evaluates instantaneous variations throughout the pedalling cycle. Consequently, increases in power output may alter the overall magnitude of asymmetry without significantly affecting the temporal shape of the NSI curve. This highlights the importance of combining both scalar and vectorial analyses to obtain a comprehensive understanding of asymmetry in cyclic movements.

Intra-subject variability also plays a role, particularly since the participants were not professional cyclists. High levels of both inter- and intra-subject variability can limit the sensitivity of SPM to detect whether observed changes in NSI patterns result from differences in power output or simply reflect natural variation. Furthermore, the protocol was designed to minimise fatigue in order to isolate the effect of power output; however, fatigue is known to substantially influence joint moment patterns (Bini and Diefenthaeler, 2010), and its absence may have moderated the expected effects.

Certain joint moments demonstrate particular sensitivity to workload. In the sagittal plane, hip and knee flexor moments show reduced asymmetry at higher power outputs, as reflected in *ADC*, aligning with previous findings (Pouliquen et al., 2018). Similarly, increases in *CCC*_*max*_ suggest improved similarity between the dominant and non-dominant limb patterns. Outside the sagittal plane, ankle inversion moments also exhibited increased *CCCmax* with higher workloads, indicating enhanced bilateral coordination.

Finally, $\tau_{lag}$ for hip adduction moments responded to higher power by shifting positively, suggesting that the non-dominant leg began to lead the dominant leg. Similar trends, although not statistically significant, were observed for knee adduction and internal rotation moments of the hip and knee, potentially reflecting a reduced capacity to coordinate complex multi-planar movements under greater effort, a phenomenon reported in previous studies (Pouliquen et al., 2018; Nikodelis et al., 2005; Martín-Sosa et al., 2022).

The results obtained in this study suggest that, in cases where pedalling asymmetries are observed, it would be advisable to temporarily increase pedalling power in order to minimise the level of asymmetry. This increase in power should be accompanied by verbal and visual feedback to enhance the effectiveness of the pedalling technique readjustment (Bini et al., 2017; Kell and Greer, 2017; Murray, 2023).

### 4.4 Initial hypothesis evaluation

At the outset of this study, four hypotheses were formulated. The first posited that asymmetries would be present across all three anatomical planes, not solely in the sagittal plane. This hypothesis stems from the traditional two-dimensional approach typically applied in cycling biomechanics, where analyses are often restricted to movements and joint moments occurring in the sagittal plane. Although the largest range of motion does indeed occur in this plane, several studies have highlighted the biomechanical relevance of movements and joint moments in the frontal and transverse planes, particularly in the context of injury prevention and overload management. In light of the results obtained, both at the global and individual levels, the first hypothesis can be accepted.

The second hypothesis highlighted the importance of examining asymmetries at a vectorial rather than a purely scalar level. Vectorial analysis allows the temporal evolution of asymmetries to be captured, revealing whether they persist throughout the entire pedalling cycle or are confined to specific phases. This approach provides a more comprehensive understanding of asymmetry dynamics, supporting the validity of the second hypothesis.

Moreover, the use of the NSI, a vectorial variable, alongside scalar variables such as the *CCC*_*max*_ and $\tau_{lag}$ , enabled the identification of the mechanical origins of the asymmetries, which could not be attributed solely to differences in magnitude between patterns. This evidence allows for the acceptance of the third hypothesis.

Finally, regarding the fourth hypothesis, whether power output influences asymmetries, although the results did not fully align with initial expectations, the analysis nonetheless indicated that asymmetries are indeed affected by changes in power output. Therefore, the fourth and final hypothesis proposed at the beginning of this study can also be accepted.

### 4.5 Limitations

The interpretation of these findings must be considered in the context of several methodological limitations inherent to kinetic cycling studies. A primary challenge was the high inter-subject variability observed, a common phenomenon in cycling biomechanics, which contributed to the lack of statistically significant differences in the continuous NSI evolution (SPM analysis). Such variability suggests that motor control strategies for compensating asymmetry are highly individualized. Despite this variability limiting the statistical power to detect subtle workload effects on the continuous pattern, the vectorial approach using *CCC*_*max*_ and $\tau_{lag}$ allowed robust trends in bilateral coordination to be identified.

Future research will consider the application of linear mixed-effects models (LMMs) as an alternative to traditional repeated-measures ANOVA. Mixed models offer greater flexibility in handling complex data structures by incorporating both fixed effects (e.g., experimental conditions) and random effects (e.g., subject-specific variability). This approach would allow for more accurate modelling of inter- and intra-subject variability, particularly in datasets with unequal measurement intervals.

Another limitation is related to the study sample, which consisted of 10 male amateur cyclists, consistent with previous research in the field (Mornieux et al., 2007; Carpes et al., 2007b; Bini and Diefenthaeler, 2010; Bini et al., 2016; Pouliquen et al., 2018; Bini, 2021; Pouliquen et al., 2021; Martín-Sosa et al., 2025). The participant selection was restricted to this homogenous group to isolate the effects of fatigue on the variables of interest and to avoid confounding factors related to gender or performance level. Consequently, the generalizability of the results to female or professional cyclists is limited. Future research should extend the analysis to different populations to determine whether power output affects male and female, as well as amateur and professional cyclists, in comparable ways.

The protocol was designed to isolate the effect of power output without inducing fatigue, ensuring that the measured asymmetries reflected baseline motor control strategies rather than compensations arising from fatigue. While this approach strengthens the interpretability of the observed patterns, it limits the extrapolation of findings to high-performance or endurance scenarios. Interpretation is further constrained by the absence of an established 3D asymmetry index “gold standard” in cycling research. Although the combination of the modified NSI, *CCC*_*max*_, and $\tau_{lag}$ provides a comprehensive vectorial assessment, direct comparison with prior studies using scalar metrics (e.g., peak ratios) remains challenging.

## 5 Conclusion

The primary objective of this study was to analyse the presence and characteristics of asymmetries in the three-dimensional joint moments of the lower limbs during cycling, by employing a new approach that combines a modification of NSI, *CCC*_*max*_ and $\tau_{lag}$ and the *ADC*. A secondary objective was to evaluate the effect of cycling at different power outputs on the development of these asymmetries.

Firstly, a revision of the NSI proposed Gouwanda and Senanayake (2011) was carried out to optimise its performance for assessing asymmetries in highly dissimilar joint moment patterns. This revision involved a modification in the normalisation process of the variables under study. A comparative analysis was conducted for all participants using both the original NSI and the modified version proposed in the present study. The results demonstrated that while the original NSI exhibited limitations in certain exceptional cases, the revised NSI consistently yielded coherent and interpretable results.

The global analysis of asymmetries across the 10 participants revealed substantial variability. This variability is primarily attributable to the absence of a gold standard for the temporal evolution of asymmetries, regardless of the pedalling proficiency of the participants. This limitation precluded meaningful aggregate analyses and necessitated the selection of a representative subject for in-depth individual analysis.

The detailed analysis of the chosen participant enabled several key conclusions. First, the use of the NSI, a vectorial variable, allowed for the characterisation of the temporal evolution of asymmetries across the pedalling cycle, identifying periods of peak and minimal asymmetry, as well as whether these values remained above or below a defined threshold over time. Secondly, the scalar variables *CCC*_*max*_ and $\tau_{lag}$ provided insight into the similarity between joint moment patterns and the temporal offset between them. This analysis confirmed the presence of asymmetries across all anatomical planes, with the hip consistently displaying the highest degree of asymmetry.

However, it was the combined use of NSI, *CCC*_*max*_ and $\tau_{lag}$ that enabled the mechanical origins of the asymmetries to be elucidated. In some cases, asymmetries were attributable to mere timing discrepancies in joint moment application, despite the patterns being highly similar and nearly identical in magnitude. Conversely, other cases revealed that asymmetries arose solely due to differences in moment magnitudes, despite the patterns being in phase and morphologically similar. Additionally, some asymmetries appeared to stem from a combination of both causes or from complete temporal and morphological divergence between the joint moment patterns.

The integrated use of these coefficients holds considerable potential for application in cycling at all levels, from amateur to professional. As documented in the literature, joint moments are implicated in a range of overuse injuries and musculoskeletal overloads (Shen et al., 2018). Therefore, the identification of asymmetries in these variables, and, crucially, an understanding of their mechanical origins, could contribute to injury prevention and performance optimisation.

Finally, the effect of cycling at different power outputs on asymmetry was investigated using both SPM and conventional ANOVA methodologies. At the vectorial level, no statistically significant differences were found, likely due to the combined effects of high intra- and inter-subject variability and the absence of a gold standard for temporal asymmetry profiles. In contrast, the ANOVA analysis revealed statistically significant effects, with asymmetries tending to decrease as power output increased in those cases where statistical significance was obtained.

## Data Availability

The original contributions presented in the study are included in the article/Supplementary Material, further inquiries can be directed to the corresponding author.
